# Metformin combined with endoscopic therapy in patients with familial polyposis associated with carcinoma: A case report

**DOI:** 10.1097/MD.0000000000032408

**Published:** 2022-12-23

**Authors:** Dong-Jie Sun, Xiao-Jian He, Hai-Tao Li, Bao-Xiang Luo, Lin-Xin Zhou, Xiang-Peng Zeng, Da-Zhou Li, Wen Wang

**Affiliations:** a Department of Digestive Diseases, Fuzong Teaching Hospital of Fujian University of Traditional Chinese Medicine, Fuzhou, China; b Department of Digestive Diseases, The Fuzong Clinical Medical College, Fujian Medical University, Fuzhou, China; c Department of Digestive Diseases, 900th Hospital of PLA, Fuzhou, China; d Department of Digestive Diseases, Oriental Hospital Affiliated to Xiamen University, Fuzhou, China.

**Keywords:** colon polyp, endoscopic therapy, familial adenomatous polyposis, metformin

## Abstract

Familial adenomatous polyposis (FAP) is an autosomal dominant genetic disease, with a very high cancer rate. At present, endoscopic resection of polyps ≥ 1 cm is often chosen for patients with non-cancerous polyps who are unwilling to undergo surgery, and regular review is conducted. Once the polyps are pathologically confirmed to be cancerous, surgical resection of the diseased large intestine is generally recommended, but surgery often leads to a series of complications. So what do you do with cancer patients who don’t want surgery?

**Patient concerns:**

A 19-year-old woman presented with intermittent hematochezia with abdominal pain. A colonoscopy revealed hundreds of intestinal polyps.

**Diagnoses:**

The patient had a family history of FAP, and there were hundreds of polyps in the intestine. The pathology was adenomatous, and some polyps became cancerous, which met the diagnostic criteria of FAP.

**Interventions:**

Endoscopic examination was arranged for the patient, the resection of intestinal polyps ≥ 1 cm was given priority, and other polyps were removed as far as possible. After that, metformin 500 mg orally was given twice a day, and endoscopic follow-up was conducted every 6 months. During each endoscopic follow-up, intestinal polyps ≥ 1 cm were preferred to be removed, and other polyps were removed as far as possible.

**Outcomes:**

The patient’s abdominal pain and blood in the stool disappeared after endoscopic treatment. Cancerous polyps were found at the second and third follow-up visits, but the patient always refused surgical treatment. After 4 years of follow-up, polyp load was significantly reduced, abdominal pain and bloody stool symptoms did not appear again, and imaging examination showed no tumor recurrence and metastasis.

**Lessons:**

Endoscopic polyp resection is an important method to treat the clinical symptoms of FAP. Metformin combined with endoscopic therapy is a good alternative for patients with familial polyposis who do not want surgery. When the polyp is cancerous and the polyp is radically resected by the endoscope, if the patient refuses additional surgery, oral metformin combined with endoscopic follow-up can be considered.

## 1. Introduction

The canceration rate of familial adenomatous polyposis (FAP) is very high. The earliest age of canceration is 15 ~ 25 years old, and most of them are around 40 years old. The lifetime canceration rate is almost 100%.^[1–3]^ Surgical intervention often causes the loss of colon stool storage function, can have abdominal pain, and diarrhea symptoms, up to dozens of times a day, and nutrient absorption function is also seriously affected, and the quality of life of patients is poor.^[4–6]^ Clinically, most FAP patients who are unwilling to take surgical treatment are treated with endoscopy. Repeated resection of colorectal polyps can ensure the quality of life of FAP patients and delay the disease progression to a certain extent.^[4,7]^ When a patient’s polyp becomes cancerous, surgical treatment is generally recommended. What happens if the patient doesn’t have surgery? Metformin is a drug for the treatment of diabetes. In recent years, many studies believe that metformin can inhibit the growth of polyps and tumors. This study reported a case of endoscopy combined with metformin in the treatment of FAP.

## 2. Case report

**Figure 1.**
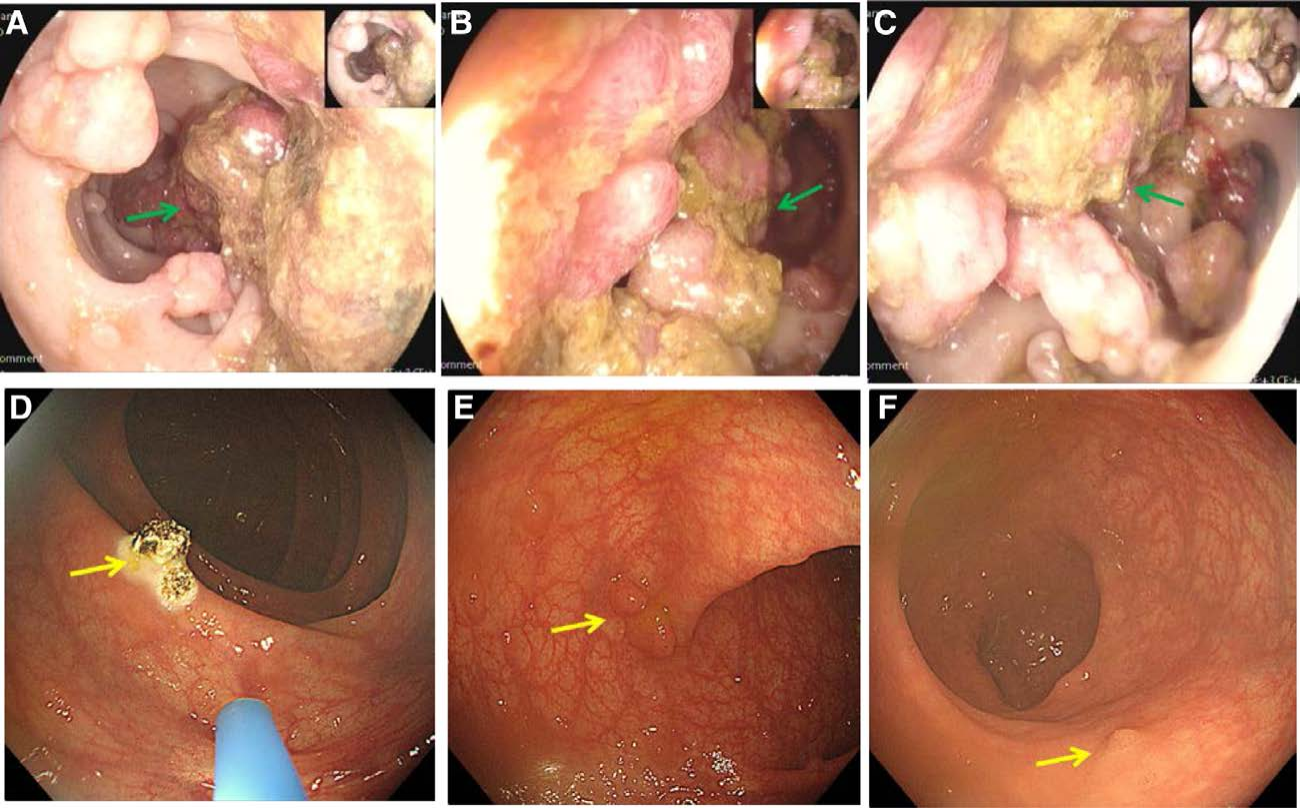
Before treatment, there were dense large polyps in the large intestine (Image a, b and c, green arrow). After endoscopic and metformin treatment, the number of polyps in the large intestine was significantly reduced at review (Image d, e and f, yellow arrow).

**Figure 2.**
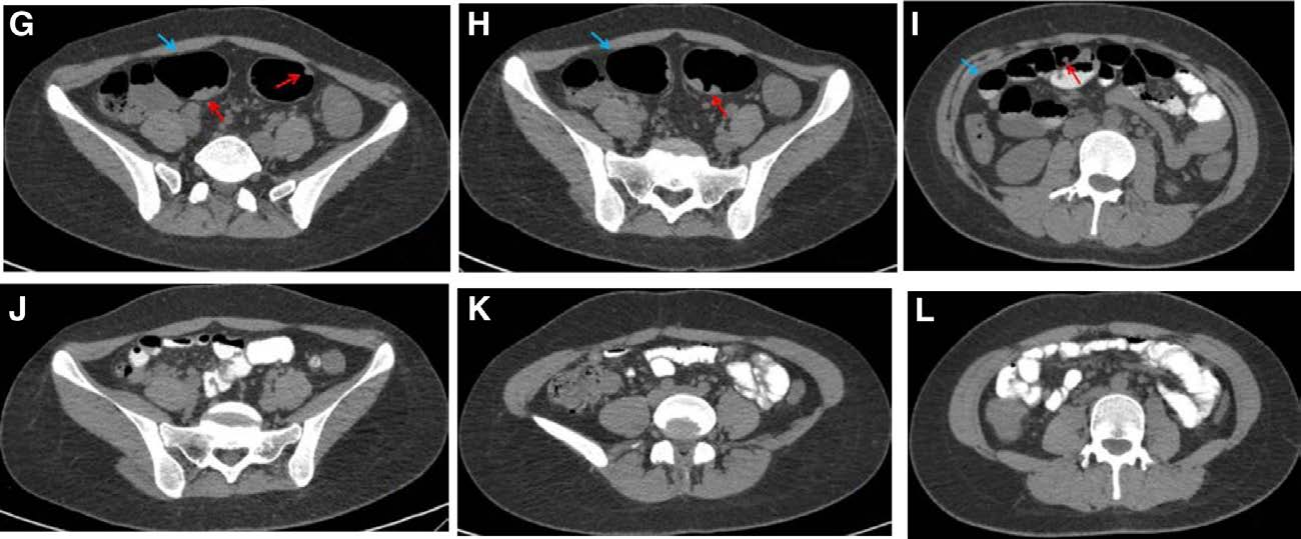
Image g, h and i indicates that abdominal CT before treatment showed significant intestinal dilatation (blue arrow), multiple polyps in colon (red arrow), and Image j, k and l indicates that no significant abnormalities were observed in CT after treatment.

A 19-year-old female was treated in our hospital in October 2018 because of “intermittent hematochezia with abdominal pain for 4 days and colonic polyps for 1 day.” The patient had intermittent hematochezia without obvious inducement 4 days before admission, which was soft and odorless, and the blood was mixed in stool, about 4 to 5 times/day, and the total amount was about 30 mL/day. After the meal, the upper abdomen repeated uninterrupted pain, other parts were not affected by pain, the duration was about 4 to 5 minutes, the pain level was different, and it was slightly relieved after defecation. FAP was diagnosed in the patient’s mother and maternal grandfather as well as in the elder aunt. And the polyps of her maternal grandfather and elder aunt were both cancerous and underwent surgical resection of the diseased intestinal segment. After admission, she was given antispasmodic, pain relief, hemostasis, and symptomatic treatment, the enteroscopy showed “hundreds of polyps in the colorectum" (Fig. 1a–c), and the pathology was adenomatous polyps, and the well-established CT showed “whole colon intestinal tube dilatation, more polypoid bulges seen in the intestine" (Fig. 2g–i). The patient was suggested surgical resection of the diseased intestinal segment, but the patient refused.

**Figure 3.**
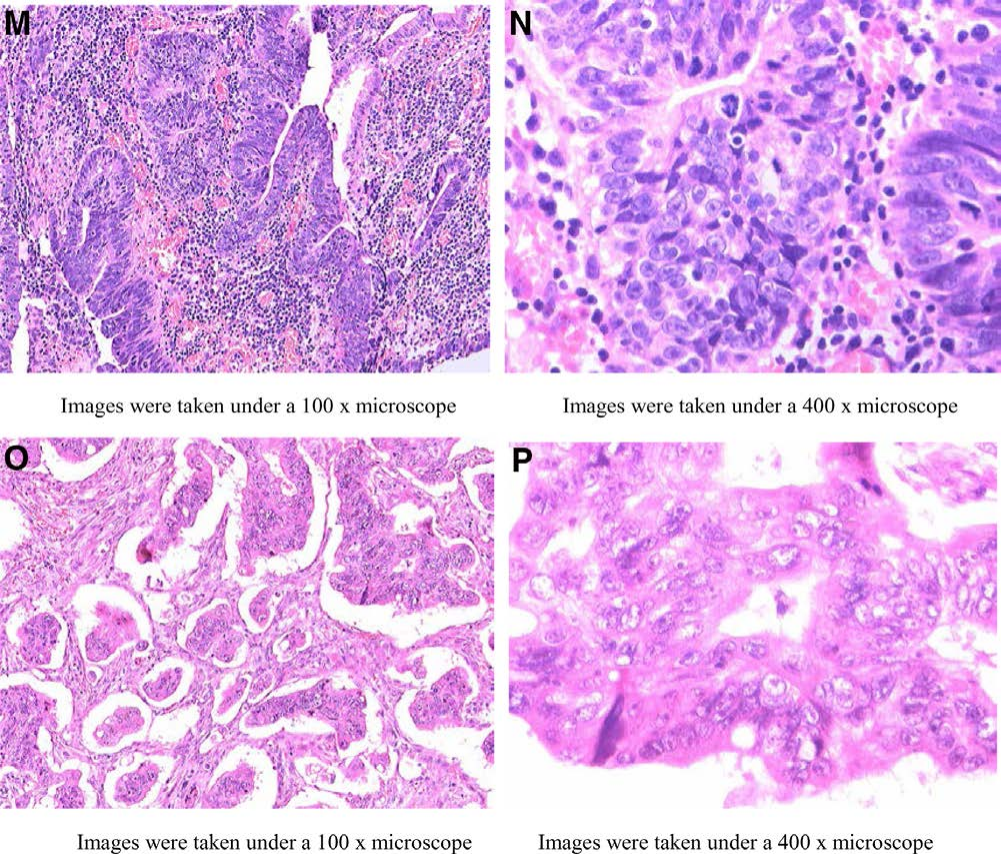
Image m and n are pathological sections obtained on November 19, 2018, which suggested tubular villous adenoma accompanied by high-grade intraepithelial neoplasia of glandular epithelium and small focal carcinomas located in the mucosa. Image o and p are pathological sections obtained on March 18, 2019. The section results indicated tubular villous adenoma with high-grade intraepithelial neoplasia and canceration of glandular epithelium, and the canceration was a moderately differentiated tubular adenocarcinoma involving submucosa.

Therefore, Enteroscopic partial polypectomy was performed. During the operation, polyps ≥ 1 cm were preferentially removed, and a total of 142 intestinal polyps were removed, among which the pathology of descending colon polyps suggested “intramucosal cancer (M2 stage)" (Fig. 3m and n), and the patient’s abdominal pain and hematochezia were relieved after surgery and the patient’s abdominal pain and hematochezia were relieved after surgery. The patient was again strongly recommended to have surgical resection of the diseased intestinal segment, but the patient still refused. Therefore, oral metformin 500mg bid Po was given to take in the meal and regular review of colonoscopy for polyp removal. The patient underwent reexamination on March 18, 2019, to remove intestinal polyps, among which the splenic polyps pathologically indicated moderately differentiated tubular adenocarcinoma (SM2 stage) (Fig. 3o and p), however, the patient refused surgical operation at all times. The patient had occasional discomforts such as abdominal distension, abdominal pain, nausea, and vomiting during medication and endoscopic treatment, but they were all relieved after symptomatic treatment, during which time the patient did not experience complications such as hypoglycemia and lactic acidosis. On October 2022, the patient came to our hospital again for partial polypectomy of the colorectum, during the operation, Colorectal polyps can be seen significantly decreased (Fig. 1d–f), review chest and abdomen enhanced CT “showed no intestinal tube dilatation and Colorectal polyps" (Fig. 2i–l), and CEA, CA199, AFP, and other tumor indicators were always normal.

## 3. Discussion

FAP is an autosomal dominant condition mostly caused by mutations in the chromosomal 5q21 region, characterized by the appearance of tens to thousands of adenomatous polyps in the digestive tract and can present with a range of clinical manifestations such as diarrhea, abdominal pain, and haematochezia. The disease has an extremely high cancer incidence, with most developing in their teens or so, and will not be avoidable without intervention.^[1–3]^ This patient needs to be differentiated from colorectal polyps and colorectal cancer. Colorectal polyps are often found by chance during colonoscopy, ranging from several to dozens, but the cancer rate is relatively low, and no obvious familial. Although many patients with colorectal cancer have family history, they will not be complicated with hundreds of polyps, and the age of onset of both colorectal polyps and colorectal cancer is generally older, while the patient in this case was young. Hundreds of colorectal polyps were found by colonoscopy, and the pathology was adenomatous. His grandfather, aunt and mother all suffered from FAP, which met the diagnostic criteria of FAP, and the patient and his family members suffered from colon cancer, which also supported this point. At present, surgery is the main way to treat FAP, but the residual intestinal segment still has the risk of polyp formation and cancer. Anastomotic stenosis and secondary desmoid fibroma were common complications after surgical treatment. In addition, surgical intervention often causes the loss of colon stool storage function, and many patients often have abdominal pain, and diarrhea symptoms, up to dozens of times a day, nutrition absorption function is also seriously affected, and some patients may even need long-term intravenous nutrition support, the quality of life of patients is poor.^[5,6]^ Most colorectal cancer develops from adenomatous polyps, and it has been reported that 25% of adenomatous polyps ≥ 10mm are likely to become cancerous. Studies suggest that patients who are unwilling to undergo surgery can be treated with annual resection of larger polyps. Endoscopic resection and regular follow-up are recommended for adenomatous polyps ≥ 10 mm in diameter.^[4]^ However, the discovery and removal of adenoma depend on endoscopy. Endoscopic removal of polyps can effectively prevent the occurrence of bowel cancer, thus delaying the time of surgery, preserving the normal physiological function of the intestine, and thus improving the quality of life of patients.^[4,7]^ The patient was a young woman who was unwilling to undergo surgical treatment, so endoscopic treatment was selected, which was in line with the current clinical practice.

The patient in this case had abdominal pain and blood in stool before treatment, and these symptoms were relieved after endoscopic treatment, indicating that these symptoms were related to polyps. These symptoms may be caused by the following reasons: when the polyp grows large enough, it obstructs the lumen, resulting in intestinal dilation and abdominal pain. By comparing the CT changes before and after treatment, we can see the intestinal dilation is obvious before treatment, and there are many polyps in the intestine, which is consistent with this inference (Fig. 2g–i). Blood in the stool is probably caused by erosion of the polyp surface, which is partly supported by the fact that patients stopped bleeding in the stool after the polypectomy. In general, endoscopic therapy is an important means to relieve the clinical symptoms of FAP.

Metformin is a derivative of the formin widely used to treat diabetes. Metformin increases insulin sensitivity and reduces serum insulin levels by reducing gluconeogenesis and improving insulin-mediated glucose uptake by liver and muscle cells. Since metformin does not directly stimulate insulin secretion, its hypoglycemia risk is small. Studies have found that metformin can potentially reduce the risk of colorectal polyps and colorectal cancer.^[8–10]^ Since the patient always refused surgical treatment, metformin combined with endoscopy was used to treat FAP. After follow-up, the number of polyps in the patient was significantly reduced, indicating that the combined treatment of metformin and endoscopy was effective for FAP to some extent.

Previous studies suggest that surgical treatment is recommended when the removed polyps in FAP patients become cancerous.^[11]^ During the follow-up period, the polyp became cancerous and was completely removed by the endoscope. Additional surgical treatment was recommended, but the patient refused and continued with oral metformin combined with endoscopic follow-up. No signs of tumor recurrence and metastasis were found by CT examination and colonoscopy in the past 2 years, and the number of polyps was significantly reduced, suggesting that if radical resection of cancerous polyps was confirmed by pathology and the patient refused additional surgery, oral metformin combined with endoscopic follow-up may be a good alternative.

## 4. Lessons

In summary, this case suggests that metformin combined with endoscopy is effective in the treatment of colorectal polyps in FAP, and is an important means to treat intestinal symptoms of FAP. Metformin combined with endoscopy is a good alternative when the radical resection of cancerous polyps is confirmed by pathology and the patient cannot accept surgery. However, the long-term efficacy of this regimen is rarely reported, so more studies are needed to confirm it.

## Author contributions

DJS, HTL, XJH, BXL, LXZ, XPZ, DZL, and WW participated in the acquisition of data; DJS, HTL, XJH, BXL contributed to the manuscript drafting; DJS, XPZ, DZL, and WW contributed to the analysis and interpretation of data, as well as revise of the manuscript for important intellectual content. All authors read and approved the final manuscript.

**Conceptualization:** Hai-Tao Li, Da-Zhou Li.

**Data curation:** Hai-Tao Li.

**Formal analysis:** Hai-Tao Li.

**Funding acquisition:** Wen Wang.

**Investigation:** Bao-Xiang Luo, Xiang-Peng Zeng.

**Methodology:** Bao-Xiang Luo, Xiang-Peng Zeng.

**Project administration:** Lin-Xin Zhou, Da-Zhou Li.

**Resources:** Xiao-Jian He, Bao-Xiang Luo.

**Software:** Xiao-Jian He, Bao-Xiang Luo, Xiang-Peng Zeng.

**Supervision:** Xiao-Jian He, Lin-Xin Zhou, Da-Zhou Li.

**Validation:** Dong-Jie Sun, Xiao-Jian He, Xiang-Peng Zeng.

**Visualization:** Dong-Jie Sun, Da-Zhou Li, Wen Wang.

**Writing – original draft:** Dong-Jie Sun, Wen Wang.

**Writing – review & editing:** Dong-Jie Sun, Wen Wang.
